# Comprehensively Exploring the Mutational Landscape and Patterns of Genomic Evolution in Hypermutated Cancers

**DOI:** 10.3390/cancers13174317

**Published:** 2021-08-26

**Authors:** Peng-Chan Lin, Yu-Min Yeh, Hui-Ping Hsu, Ren-Hao Chan, Bo-Wen Lin, Po-Chuan Chen, Chien-Chang Pan, Keng-Fu Hsu, Jenn-Ren Hsiao, Yan-Shen Shan, Meng-Ru Shen

**Affiliations:** 1 Department of Oncology, National Cheng Kung University Hospital, College of Medicine, National Cheng Kung University, Tainan 704, Taiwan; pengchan@mail.ncku.edu.tw (P.-C.L.); s98031083@mail.ncku.edu.tw (Y.-M.Y.); 2 Department of Genomic Medicine, National Cheng Kung University Hospital, College of Medicine, National Cheng Kung University, Tainan 704, Taiwan; z10609069@email.ncku.edu.tw; 3 Department of Computer Science and Information Engineering, College of Electrical Engineering and Computer Science, National Cheng Kung University, Tainan 704, Taiwan; 4 Division of General Surgery, Department of Surgery, National Cheng Kung University Hospital, College of Medicine, National Cheng Kung University, Tainan 704, Taiwan; hphsu@mail.ncku.edu.tw (H.-P.H.); n803421@mail.hosp.ncku.edu.tw (R.-H.C.); linbw@mail.ncku.edu.tw (B.-W.L.); cpc324@gmail.com (P.-C.C.); ysshan@mail.ncku.edu.tw (Y.-S.S.); 5 Department of Pharmacology, National Cheng Kung University Hospital, College of Medicine, National Cheng Kung University, Tainan 704, Taiwan; 6 Department of Obstetrics and Gynecology, National Cheng Kung University Hospital, College of Medicine, National Cheng Kung University, Tainan 704, Taiwan; d5580@mail.ncku.edu.tw; 7 Department of Otolaryngology, National Cheng Kung University Hospital, College of Medicine, National Cheng Kung University, Tainan 704, Taiwan; hsiaojr@mail.ncku.edu.tw; 8 Institute of Clinical Medicine, College of Medicine, National Cheng Kung University, Tainan 704, Taiwan

**Keywords:** hypermutated cancer, mutational landscape, genomics evolution, tumor mutation burden

## Abstract

**Simple Summary:**

To identify potential genetic markers for evaluating hypermutated cancers, we investigated driver mutations, mutational signatures, tumor-associated neoantigens, and molecular cancer evolution in the genetic variants of 533 cancer patients with six different cancer types. Driver mutations, including *RET*, *CBL*, and *DDR2* gene mutations, were identified in the hypermutated cancers. Cancer driver mutations and mutational signatures are associated with sensitivity or resistance to immunotherapy, representing potential genetic markers in hypermutated cancers. Using computational predictions, we identified two tumor-associated neoantigens. Sequential mutations were used in a logistic model to predict hypermutated cancers according to genomic evolution. The sequential mutation order and coexisting genetic mutations were found to influence the hypermutation phenotype. Based on our observations, we developed a new concept for hypermutated cancers, whereby sequential mutations are significant for hypermutated cancers, which are mutationally heterogeneous. Through the comprehensive assessments of cancer gene panels, mutational pattern analysis was conducted as a basis for providing recommendations regarding therapeutic strategies for hypermutated cancer patients.

**Abstract:**

Tumor heterogeneity results in more than 50% of hypermutated cancers failing to respond to standard immunotherapy. There are numerous challenges in terms of drug resistance, therapeutic strategies, and biomarkers in immunotherapy. In this study, we analyzed primary tumor samples from 533 cancer patients with six different cancer types using deep targeted sequencing and gene expression data from 78 colorectal cancer patients, whereby driver mutations, mutational signatures, tumor-associated neoantigens, and molecular cancer evolution were investigated. Driver mutations, including *RET*, *CBL*, and *DDR2* gene mutations, were identified in the hypermutated cancers. Most hypermutated endometrial and pancreatic cancer patients carry genetic mutations in *EGFR*, *FBXW7*, and *PIK3CA* that are linked to immunotherapy resistance, while hypermutated head and neck cancer patients carry genetic mutations associated with better treatment responses, such as *ATM* and *BRRCA2* mutations. *APOBEC* (apolipoprotein B mRNA editing enzyme, catalytic polypeptide-like) and DNA repair defects are mutational drivers that are signatures for hypermutated cancer. Cancer driver mutations and other mutational signatures are associated with sensitivity or resistance to immunotherapy, representing potential genetic markers in hypermutated cancers. Using computational prediction, we identified *NF1* p.T700I and *NOTCH1* p.V2153M as tumor-associated neoantigens, representing potential therapeutic targets for immunotherapy. Sequential mutations were used to predict hypermutated cancers based on genomic evolution. Using a logistic model, we achieved an area under the curve (AUC) = 0.93, accuracy = 0.93, and sensitivity = 0.81 in the testing set. The sequential patterns were distinct among the six cancer types, and the sequential mutation order of *MSH2* and the coexisting *BRAF* genetic mutations influenced the hypermutated phenotype. The *TP53*~*MLH1* and *NOTCH1*~*TET2* sequential mutations impacted colorectal cancer survival (*p*-value = 0.027 and 0.0001, respectively) by reducing the expression of *PTPRCAP* (*p*-value = 1.06 × 10^−6^) and *NOS2* (*p*-value = 7.57 × 10^−7^) in immunity. Sequential mutations are significant for hypermutated cancers, which are characterized by mutational heterogeneity. In addition to driver mutations and mutational signatures, sequential mutations in cancer evolution can impact hypermutated cancers. They characterize potential responses or predictive markers for hypermutated cancers. These data can also be used to develop hypermutation-associated drug targets and elucidate the evolutionary biology of cancer survival. In this study, we conducted a comprehensive analysis of mutational patterns, including sequential mutations, and identified useful markers and therapeutic targets in hypermutated cancer patients.

## 1. Introduction

All cancers result from the accumulation of genetic mutations. Hypermutated cancers, constituting 5–10% of all cases of cancer, are characterized by a high tumor mutational burden (TMB), which is a useful biomarker for predicting the immune checkpoint inhibitor (ICI) response in cancer patients [1]. However, immunotherapies have exhibited variable efficacy in human malignancies [2]. More than 50% of hypermutated cancer patients fail to respond to immunotherapy. Some cancer subtypes still have a poor response to therapy that cannot be predicted by TMB. Intra- or intertumor heterogeneity may contribute to inferior clinical outcomes and drug resistance in hypermutated cancers [3]. Comprehensively exploring the mutational landscape and genomic evolution of cancers can provide insight into the biology of tumor heterogeneity.

By profiling the genomes of patients with high TMB, we found that positivity for microsatellite instability-high (MSI-H) and programmed death-ligand 1 (PD-L1) is uncommon in some cancer patients [4,5]. Beyond MSI and PD-L1 expression, there is a need to identify additional emerging driver mutation biomarkers, mutational signatures, and tumor-associated neoantigens in hypermutated cancers to guide immunotherapy. In previous studies, genomic alterations (such as those that lead to changes in *STK11*, *EGFR*, *JAK*, or *APOBEC* activity) were associated with a response or resistance to immunotherapy [6,7]. In addition to the driver mutations present in each patient, mutational signatures are helpful for understanding the specific mutagenesis process of hypermutated cancers. Hypermutated cancers develop as a consequence of extrinsic or intrinsic factors, such as tobacco smoke, ultraviolet light, apolipoprotein B mRNA editing enzyme (APOBEC, catalytic polypeptide-like) dysfunction, or DNA repair gene mutations. Mutational heterogeneity in molecular mechanisms is observed across hypermutated cancer types [8].

Understanding the sequence of genetic mutations can be used to explore tumor heterogeneity in hypermutated cancers. Herein, the traditional hallmarks of cancer are discussed, as one mutation drives a particular phenotype. However, the order and coexistence of sequential mutations are also critical in tumor biology. In a previous study [9], the acquisition order of *KRAS* and *TP53* gene alterations led to differential adrenocortical tumor phenotypes. In the model of cancer evolution featuring sequential mutations, the order of coexisting mutations influences cancer behavior in hypermutated cancers. Here, we used cancer evolution to assess the tumor heterogeneity of hypermutated cancers. We emphasize the importance of hypermutation-associated sequential mutations. By identifying the sequence of mutations in the cancer evolution model, we can understand its clinical impact.

Comprehensively understanding hypermutagenesis holds great clinical value. The primary aim of this study is to investigate and conduct a comprehensive mutational pattern analysis to provide therapeutic strategies in hypermutated cancer patients. The mutagenesis process was investigated in both hypermutated and nonhypermutated cancer patients. By analyzing the cancer gene panel in hypermutated cancers, we can identify potential biomarkers and drug targets and provide additional biological information for hypermutated cancers.

## 2. Materials and Methods

### 2.1. Enrollment of Cancer Patients

This was a cohort study of cancer patients. Eligible cancer patients were aged ≥ 20 years with histologically confirmed pathological stage I–IV breast cancer adenocarcinoma (BRCA), stage II–III colorectal adenocarcinoma (CRC), stage II–IV endometrial cancer (EC), stage II–IV, head and neck squamous cell carcinoma (HNSC), stage I–IV ovarian cancer (OV), or stage I–V pancreatic adenocarcinoma (PDAC), as well as an Eastern Cooperative Oncology Group performance status (ECOG PS) of 0–1, and adequate organ function. The histological subtypes of endometrial cancer include endometrioid, high-grade serous, clear cell carcinoma, and others. The histological subtypes of ovarian cancer include high-grade serous, clear cell carcinoma, endometrioid, mucinous, and others. Exclusion criteria were receiving chemotherapy within six months, other malignancies, and life expectancy less than one year. A total of 533 patients with six different types of cancer, 55 with breast cancer (BRCA), 129 with colorectal cancer (CRC), 155 with endometrial cancer (EC), 67 with head and neck cancer (HNSC), 27 with ovarian cancer (OV), and 100 with pancreatic cancer (PDAC), were recruited for this study at the National Cheng Kung University Hospital (NCKUH) between January 2015 and January 2017. Follow-up continued through August 2019. Clinical information and tissue samples for DNA extraction were collected at the time of enrollment. The NCKUH institutional review board approved this study (A-ER-103-395, A-ER-104-153, and B-ER-109-154), and all participants provided written informed consent.

### 2.2. Targeted Tumor Sequencing Using a Cancer Gene Panel

A total of 533 primary tumor samples were sent for histologic assessment, followed by the extraction of nucleic acids from formalin-fixed paraffin-embedded blocks at NCKUH. Specimens were reviewed by pathologists, who determined the percentage of viable tumor nuclei and the adequacy of samples for mutational profiling. Deep targeted sequencing of tumor samples was performed by Oncomine Comprehensive Assays (OCA) (Thermo Fisher Scientific, Waltham, MA, USA). All samples were analyzed using Torrent Suite Software 5.0.4 (Thermo Fisher Scientific, Waltham, MA, USA), aligning all reads to the hg19 reference genome. Variant calling was performed using the Torrent Variant Caller plugin (Thermo Fisher Scientific, Waltham, MA, USA) version 5.0.4.0. The Oncomine cancer panel was validated to measure tumor mutation burden in routine molecular diagnostics and according to the TMB Harmonization Project [10,11]. To verify the OCA, we performed a verification test using commercially available control reference agents of Structural Multiplex Reference Standard gDNA and Quantitative Multiplex Reference Standard (Horizon Diagnostics, Cambridge, United Kingdom). Next-generation sequencing (NGS) reactions were performed using three replicates to validate control testing. The laboratory-developed tests were approved by the Taiwan Food and Drug Administration (TFDA-LDTs) and accredited by the Taiwan Accreditation Foundation (TAF). We ensured preanalytical quality control with a DNA concentration > 10 ng/µL and library concentration ≥ 100 pM. For higher detection sensitivity and quality control of genomic sequence variations, the required average target coverage was more than 1000×.

### 2.3. Immune Response Gene Expression Data

Cancer tissues with immune response gene expression data were obtained from 99 CRC patients. RNA was prepared from formalin-fixed paraffin-embedded (FFPE) tissue that was extracted using the RecoverAll Total Nucleic Acid Isolation Kit (Thermo Fisher Scientific). RNA concentration was determined on an Invitrogen™ Qubit™ Fluorometer using the Qubit™ RNA High Sensitivity Assay (Thermo Fisher Scientific). Twenty nanograms of RNA were used for each reverse transcription reaction, and cDNA was prepared using the SuperScript™ IV VILO™ Master Mix Kit. Immune response libraries were prepared using the Ion AmpliSeq™ Kit for Chef DL8 with the Ion Chef™ System according to instructions in the Oncomine™ Immune Response Research Assay user guide (Pub. No. MAN0015867). Raw gene expression data were preprocessed using Torrent Suite (Thermo Fisher Scientific) and normalized using the min-max feature scaling approach.

### 2.4. Statistical Analysis

The odds ratio was used to assess the association between hypermutated and nonhypermutated groups. The oversampling method was used in the hypermutated group before the calculation of the odds ratio. A *t*-test was used to determine the association between immune expression and evolutionary trajectories. Kaplan–Meier curves were used to evaluate recurrence-free survival (RFS), which was defined as the time between surgery and cancer recurrence. A *p*-value adjusted by a false discovery rate (FDR) or a Bonferroni (BF) critical value < 0.05 was considered statistically significant. All statistical analyses were performed in the R environment.

### 2.5. Cancer Driver Mutation Spectra and Mutational Signature Analysis

Many studies have demonstrated filtering of common germline polymorphisms from tumor-only NGS data through population frequency [12]. Variant allele frequencies lower than 5% were filtered out to exclude potential artifacts. To identify potential cancer driver mutations, we used the populational allele frequency cutoffs of 1% with respect to Taiwan Biobank and Exome Aggregation Consortium-East Asian (ExAC-EAS) databases [13,14]. The minimal read depth threshold is >50 in this study. Assuming we have a variant with a 5% variant allele frequency, at least three reads with alternative alleles would be guaranteed. Exonic single-nucleotide variants (SNVs) were divided into 96 trinucleotides with 16 possible flanking nucleotide contexts. Each patient with >20 exonic genetic variants was analyzed for a personal mutational signature [15,16]. The Catalog of Somatic Mutations in Cancer (COSMIC; known mutational signatures) was analyzed using the R package deconstructSigs [17].

### 2.6. Evolutionary Tree Construction Trajectory Analysis

We used targeted tumor sequencing for cancer evolution. Cancers evolve as sequential clone and subclone driver mutations [18,19]. All genetic SNVs and small insertions and deletions (indels) were used to build evolutionary trees for each patient. A clonal inference analysis for each patient was performed using the R package SciClone [20]. Evolutionary trees and repeated evolutionary trajectories of sequential mutations were produced by the R package Revolver [21].

### 2.7. Machine Learning Model and Analysis

#### 2.7.1. Selecting Evolutionary Trajectories for the Hypermutation Prediction Model

We used the least absolute shrinkage and selection operator (LASSO) regression algorithm for feature selection. We built the prediction model using six machine learning methods: AdaBag (bagging), AdaBoost (boosting), C5.0, logistic regression (LR), random forest (RF), and support vector machine (SVM). We divided our dataset into a training set (80%) and a testing set (20%). Feature selection, oversampling, and classifiers were implemented using the R packages glmnet [22], ROSE [23], and caret [24].

#### 2.7.2. Identification of Putative Neoantigens

We simulated the HLA distribution of the Taiwanese Han Chinese population from the HLA database [25]. Neoantigens were defined as peptides of approximately 9–11 amino acids in length with a rank of less than 2%. Strong binding affinity neoantigens were defined as those with a percentage less than 0.5%. We used the tool NeoPredPipe (Neoantigen Prediction Pipeline) [26] to predict neoantigens from patient genetic mutations and HLA data.

## 3. Results

### 3.1. Identification of Potential Cancer Driver Mutations in Hypermutated Cancers

**Figure 1:**
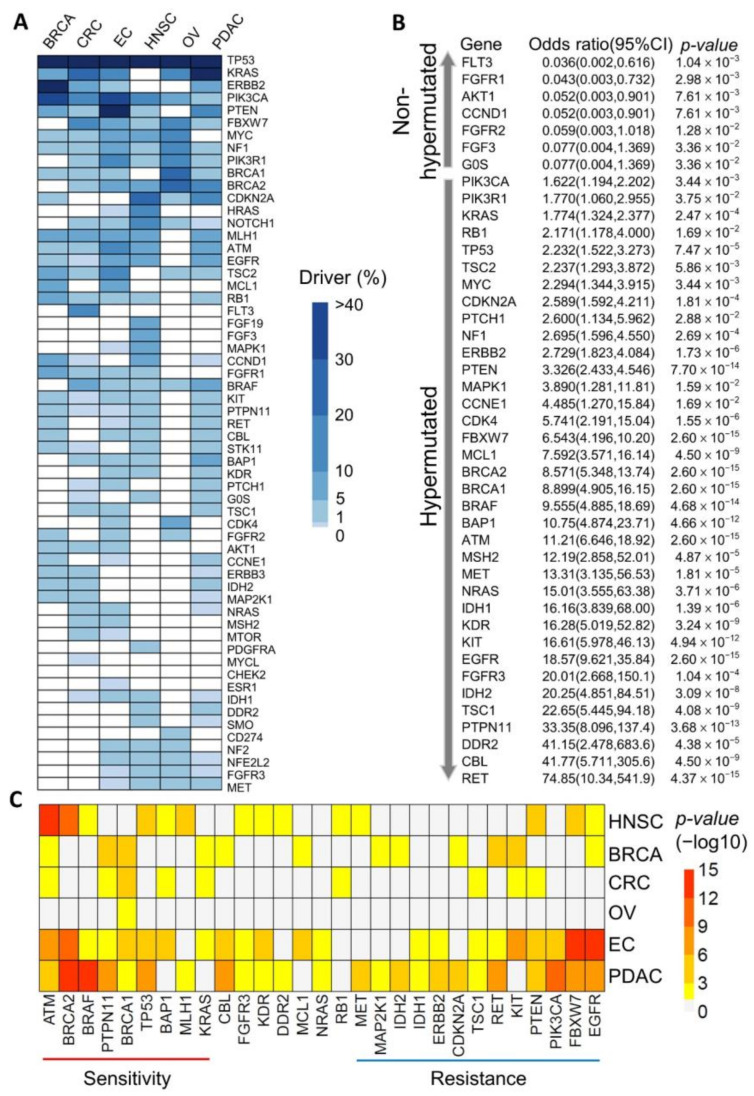
Driver mutations in hypermutated cancers. (**A**) The spectra and percentages of driver mutations in six cancer types: breast (BRCA), colorectal (CRC), endometrial (EC), oral (HNSC), ovarian (OV), and pancreatic (PDAC). *ERBB2* driver mutations were the most common mutations in BRCA; *KRAS* driver mutations were the most common in PDAC and CRC; *BRCA1* and *BRCA2* driver mutations were the most common in OV; *CDKN2A* and *HRAS* driver mutations were the most common in HNSC, and *PTEN* genetic mutations were the most common in EC. (**B**) Genetic driver mutations in all 533 patients with hypermutated and nonhypermutated cancers. *p*-values were calculated using an odds ratio with a false discovery rate (FDR) correction, and a *p*-value < 0.05 was considered statistically significant. There are seven genes with odds ratios < 1 in patients with nonhypermutated cancers. The other genes have values > 1 in patients with hypermutated cancers. (**C**) Mutation-associated sensitivity or resistance to immunotherapy. Heatmap of hypermutation-associated genetic driver mutations in six cancer types. Genes that were significant in at least two cancer types are shown. Most hypermutated EC and PDAC patients carried *EGFR*, *PIK3CA*, and *FBXW7* genetic mutations, and most hypermutated HNSC, EC, and PDAC patients carried the *ATM*, *BRRCA2*, and *BRAF* genetic mutations. The *p*-value was calculated by an odds ratio and adjusted by FDR correction.

To investigate driver mutations in hypermutated cancers, we explored the genetic mutation profiles of 533 cancer patients with six cancer types. We annotated potential driver targets in cancer patients using genetic variants from the Oncomine reporter. A total of 115 genes and 7555 genetic variants were identified in 533 samples. As shown in Figure 1A and Table S1, we identified the spectrum of driver mutations in patients with six different cancer types. Except for the *TP53* genetic mutations, which were found in all cancer types, we observed the highest percentages of mutations in *ERBB2* and *PIK3CA* in breast cancer, *KRAS* and *PIK3CA* in colorectal cancer, *PTEN* and *PIK3CA* in endometrial cancer, *CDKN2A* and *HRAS* in oral cancer, *BRCA1* and *BRCA2* in ovarian cancer, and *KRAS* and *CDKN2A* in pancreatic cancer. The top 15 genetic variants from each cancer tissue are shown in Figure S1. We evaluated driver mutations in cancer patients after stratification by hypermutation status. There were more *FLT3*, *FGFR1*, and *AKT1* mutations (*p*-value < 0.05) in nonhypermutated cancers, while there were more *RET*, *CBL*, and *DDR2* mutations (*p*-value < 0.05) in hypermutated cancers (Figure 1B and Table S2). We found that the frequency of genetic mutations was an important mutation feature in cancers, and frequent mutation was also notable in The Cancer Genome Atlas (TCGA) samples. The cosine similarity of gene frequencies between our cancer study and TCGA datasets (National Cancer Institute’s Genomic Data Commons Data Portal) was more than 95% in HNSC and PDAC and 92% in OV. The similarity was more than 85% in CRC and BRCA (Table S3). Compared with TCGA datasets, the frequencies of *KRAS* mutations in pancreatic cancer were 56.8% and 56%, respectively. In a previous study, we also found a higher frequency of *KRAS* mutations in the hypermutated group [27,28]. For specific histology types, we used Fisher’s exact test to analyze the association between driver mutations and hypermutation in endometrial and ovarian cancers. The results indicated that most hypermutated endometrioid-type EC patients carried *MCL1*, *FBXW7*, *BAP1*, and *EGFR* driver mutations (*p*-value < 0.05). No statistical significance was derived in the other histological types of endometrial cancer and ovarian cancer (Table S4). *RET*, *CBL*, and *DDR2* driver mutations might represent potential response markers in hypermutated cancers. Mutations associated with sensitivity or resistance to immunotherapy in different cancer types [7,8] are shown in Figure 1C and Table S5. Most hypermutated EC and PDAC patients carry genetic *EGFR*, *PIK3CA*, and *FBXW7* mutations, which are markers of resistance to immunotherapy. The most hypermutated HNSC, EC, and PDAC patients carry *ATM*, *BRRCA2*, and *BRAF* genetic mutations linked to immunotherapy sensitivity [7,8]. These results imply that there are heterogeneous types of driver mutations in hypermutated cancers. Cancer driver mutations are associated with sensitivity or resistance to immunotherapy, representing potential genetic markers in hypermutated cancers.

### 3.2. Mutational Signatures as Genetic Makers for Immunotherapy

**Figure 2:**
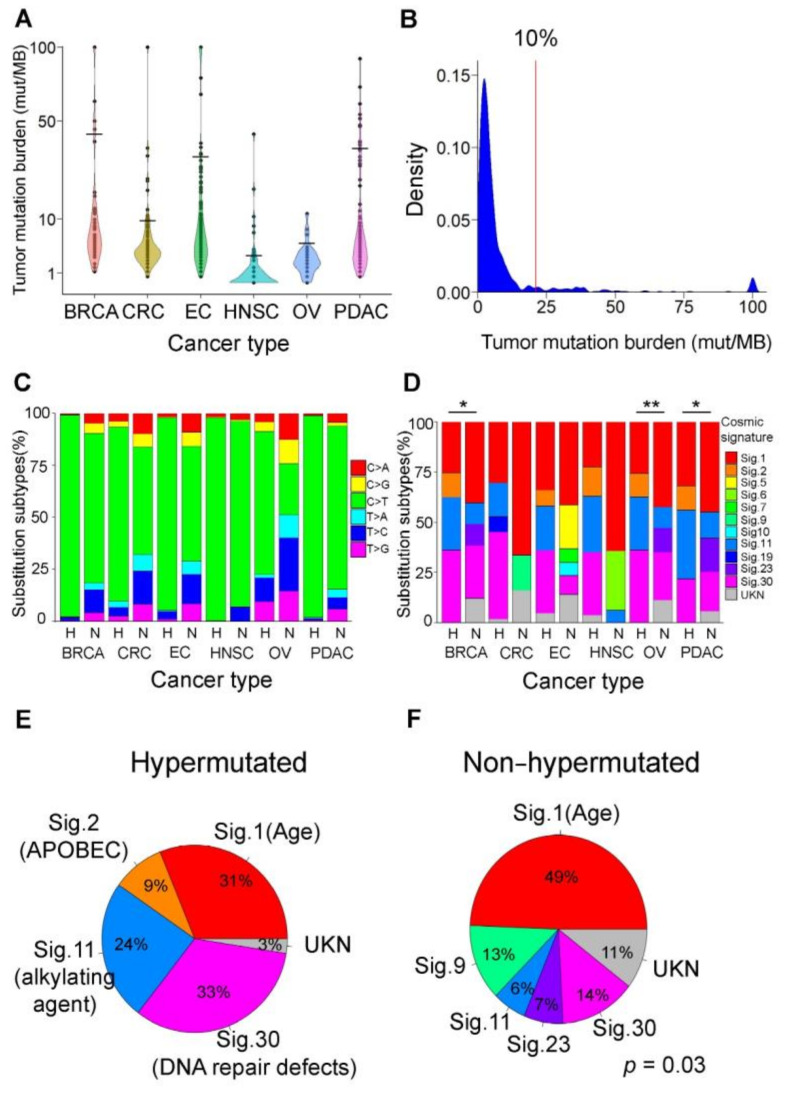
Mutational signatures in hypermutated cancers. (**A**) Violin plot of tumor mutation burden (TMB; number of exonic mutations per megabase) in 533 patients with six different cancer types. The analyzed cohort comprises 55 breast cancer, 129 colorectal cancer, 155 endometrial cancer, 67 oral cancer, 27 ovarian cancer, and 100 pancreatic cancer patients. Patients with TMB values over 100 mutations per megabase (mut/Mb) are labeled 100 mut/Mb. The cutoffs for the highest 10% TMB values in each cancer type are 4.84, 9.53, 35.72, 42.79, 31.83, and 2.87 mut/Mb in ovarian, colorectal, pancreatic, breast, endometrial, and oral cancer, respectively. The TMB data differ for different cancer types. (**B**) Density plot of TMB in all cancer patients. The plot shows the mutation rates across our cohort, and the red line shows the cutoff of the highest 10% TMB values with 21 mut/Mb. (**C**) Frequency of substitution subtypes for six cancer types. Hypermutated (H) and nonhypermutated (N) cancers. Most single-nucleotide mutation substitutions were C > T in cancer tissue. (**D**) Bar plot of the mutational signature in six cancer types for hypermutated. * *p*-value < 0.05 and ** *p*-value < 0.01 (H) and nonhypermutated (N) cancers. The percentage of age-related signature 1 in hypermutated cancers is lower than that in nonhypermutated cancers. There were more signatures related to the *APOBEC* family of cytidine deaminases in hypermutated cancers than in nonhypermutated cancers (*p*-value < 0.05). (**E**) Pie chart of mutation signatures for hypermutated cancers. Mutation signatures were constructed, and a higher percentage of signature 11 (alkylating agents) and signature 30 (DNA repair defects) compared to signature 2 (*APOBEC* family of cytidine deaminase) is found in hypermutated cancers compared to nonhypermutated cancers (*p*-value = 0.03 by chi-square test). (**F**) Pie chart of mutation signatures for nonhypermutated cancers. Mutation signatures were constructed, and a higher percentage of signature 1 (age-related) was found in nonhypermutated cancers than in hypermutated cancers.

In addition to driver mutations, mutational signatures are helpful for understanding the specific mutagenesis process of hypermutated cancers. In a previous study, and in our results (Figure S2), panel-based tumor mutational burden (TMB) values suggest a biomarker when compared with the results of whole-exome sequencing (WES) in clinical practice [29]. To understand the mutation load distribution of our cohort, we calculated the mutation rates of 533 patients with six different cancer types (Figure 2A). Here, we defined patients with a hypermutation status in each cancer type as those in the top 10% of each cancer group according to the definition of hypermutation in a previous study [8]. There was a higher tumor mutation burden in breast (43 mutations per megabase (mut/Mb)), endometrial (32 mut/Mb), and pancreatic (36 mut/Mb) cancers, while the TMB values in oral cancer and colorectal cancer were 2.87 and approximately 10 mut/Mb, respectively. We also showed that the density plot in our cohort had a long tail distribution (Figure 2B), which matches the distribution observed in a previous study [30]. When analyzing the single nucleotide substitutions of all genetic mutations (Figure 2C), most single nucleotide mutation substitutions showed C > T in hypermutated cancers. There was no statistically significant difference in the proportion of mutation substitutions within the six cancer types. We deconstructed the mutational signatures in all cancer samples (Figure 2D, * *p*-value < 0.05, Figure S3). There were statistically significant differences among BRCA, OV, and PDAC patients. We found that approximately 31%, 9%, 24%, and 33% of the mutational signatures of hypermutated cancers consisted of signature 1 (age-related), signature 2 (APOBEC-related), signature 11 (alkylating agent-related) and signature 30 (DNA repair defect-related), respectively (Figure 2E); while 49%, 13%, 6%, 7%, and 14% of the mutational signatures of nonhypermutated cancers consisted of signature 1, signature 9, signature 11, signature 23, and signature 30, respectively (Figure 2F, *p*-value = 0.03 by chi-square). Mutational signatures related to *APOBEC*, an alkylating agent and DNA repair defect, appeared to play a strong role in hypermutated cancers, whereas the age-related mutational signature was more prominent in nonhypermutated cancers. Beyond the cancer driver mutations, DNA repair defects and *APOBEC* mutational signatures are good immunotherapy markers. These results indicate that there are different mutagenesis processes between hypermutated and nonhypermutated cancer patients.

### 3.3. Hypermutation-Derived Neoantigens

**Figure 3:**
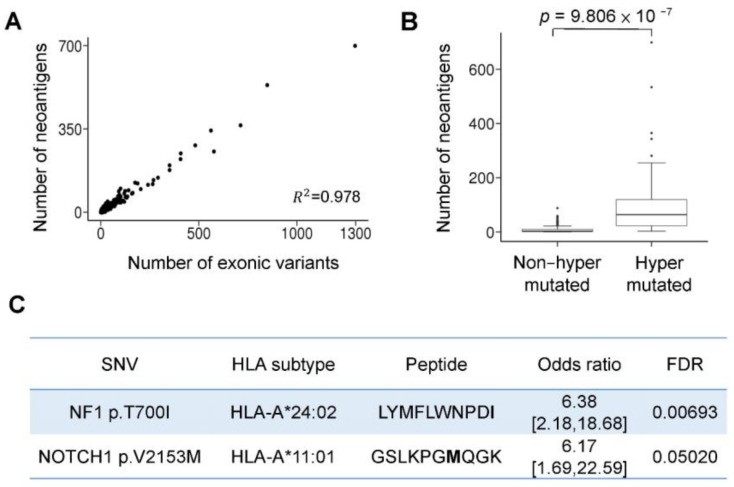
Hypermutation-derived neoantigens. (**A**) The correlation between exonic variants and the number of strongly binding neoantigens in the 533 patients. There is a strong relationship (*p-*value = 0.978 by Pearson’s correlation) between tumor mutational burden and neoantigens. (**B**) Neoantigen numbers by subgroups. The hypermutated group possesses more neoantigens than the nonhypermutated group, with median values of 64 and 3 neoantigens, respectively; *p*-value = 9.806 × 10^−7^ by *t*-test. (**C**) Neoantigens in the hypermutated group. Two novel neoantigens were identified, *NF1* p.T700I and *NOTCH1* p.V2153M. An odds ratio was used to determine the correlation between hypermutation and neoantigens. The *p*-value was adjusted for FDR (false discovery rate). * Separator.

We identified two tumor-associated neoantigens in hypermutated cancers from 533 patients to guide immunotherapy (Figure 3). We simulated the HLA-A, HLA-B, and HLA-C distributions of the Taiwanese Han population from the HLA database [25], and the results are shown as a bar plot (Figure S4b–d). We used the NeoPredPipe tool [26] to predict neoantigens with information about the patients’ genetic variants using a cancer panel and the simulated HLA data (Figure S4a). The number of neoantigens ranged from 0 to 699, and the exonic variants were positively correlated with the neoantigens with a strong binding affinity (*p-*value = 0.978, Figure 3A, and Table S6). The numbers of neoantigens ranged from 0 to 88 (median, 3) and 3 to 699 (median, 64) in the nonhypermutated and hypermutated groups, respectively, the difference between the two was significant (*p*-value = 9.806 × 10^−7^, Figure 3B and Table S7). These observations indicate that tumor mutational burden is correlated with neoantigen load, which is a biomarker for cancer immunotherapy. We also identified two neoantigens with a strong binding affinity that were associated with hypermutation (Figure 3C). The binding affinities of *NF1* p.T700I and *NOTCH1* p.V2153M neoantigens were 60.7 and 34.7 nM, respectively. The percentage of *NF1* p.T700I neoantigens present in hypermutated and nonhypermutated patients were 10% and 1.9%, respectively. The percentage of the *NOTCH1* p.V2153M neoantigen present in hypermutated and nonhypermutated patients was 7.2% and 1.3%, respectively. The *NF1* p.T700I and *NOTCH1* p.V2153M neoantigens may represent potential targets for immunotherapy in hypermutated cancer patients.

### 3.4. Sequential Mutation Clustering and Trajectory Analysis in the Evolution of Hypermutated Cancers

**Figure 4:**
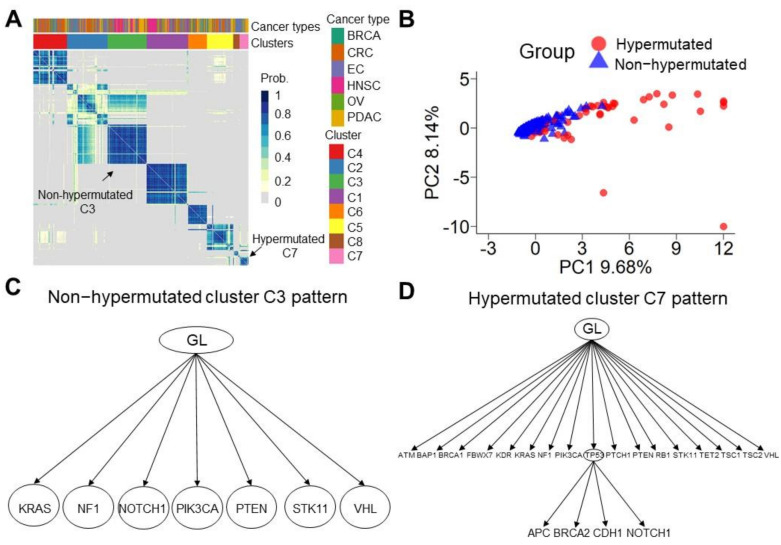
Sequential mutation clustering and trajectory analysis. (**A**) Coclustering heatmap of 533 patients with sequential mutations. A darker blue color indicates a higher probability of clustering together across each resample. Most hypermutated cancer patients are in C2, C7, and C8. (**B**) Principal component analysis (PCA) of hypermutated and nonhypermutated cancer patients by sequential mutations. Red circles represent the hypermutated group, and purple triangles show the nonhypermutated group. (**C**) The nonhypermutated cluster 3 (C3) pattern. The sequential mutations that occurred at least ten times in C3 are plotted. The C3 pattern is composed of seven sequential mutations, including the germlines (GL)~*KRAS*, *GL*~*NF1*, *GL*~*NOTCH1*, *GL*~*PIK3CA*, *GL*~*PTEN*, *GL*~*STK11*, and *GL*~*VHL*. (**D**) The hypermutated C7 pattern. Seventeen sequential mutations that occurred at least ten times in C7 are plotted. The C7 pattern is composed of 17 sequential mutations in the first clone and 4 sequential mutations in the second clone: germline *(GL)*~*ATM*, *GL*~*BAP1*, *GL*~*BRCA1*, *GL*~*FBXW7*, *GL~KDR*, *GL*~*KRAS*, *GL*~*NF1*, *GL*~*PIK3CA*, *GL*~*TP53*, *GL*~*PTCH1*, *GL*~*PTEN*, *GL*~*RB1*, *GL*~*STK11*, *GL*~*TET2*, *GL*~*TSC1*, *GL*~*TSC2*, *GL*~*VHL*, *TP53*~*APC*, *TP53*~*BRCA2*, *TP53*~*CDH1,* and *TP53~NOTCH1*.

To understand the evolution of sequential mutations in hypermutated cancers, we analyzed the evolutionary tree of cancer using the Revolver tool [21,31,32]. Our cohort was separated into eight evolutionary groups, from cluster 1 (C1) to cluster 8 (C8) (Figure 4A). No differences were observed among the eight evolutionary groups concerning the tumor type. Most of the hypermutated patients were in C2, C7, and C8. Principal component analysis (PCA) demonstrated that the two groups of hypermutated and nonhypermutated cancer patients exhibited repeated sequential mutations (Figure 4B, PC1 (9.68%) and PC2 (8.14%)). There were distinct evolutionary trajectories in the hypermutated (Figure S5a–c, and Table S8) and nonhypermutated (Figure S5d–h) groups. For example, we compared sequential mutations in patients from C7 (hypermutated) and those from C3 (nonhypermutated) (Figure 4C,D). Patients in C7 (hypermutated) presented complex clonal structures and more sequential mutations than patients in C3 in the evolution model. Late acquisition of clonal *TP53* genetic mutations with *APC*, *BRCA2*, *CDH1*, and *NOTCH1* mutations was observed in the hypermutated group (Figure 4D). There were 17 and 7 sequential mutations in the C7 and C3 groups, respectively. These results demonstrate that there are distinct sequential patterns of tumor evolution between hypermutated and nonhypermutated cancer patients (Figure S5).

### 3.5. Machine Learning Models to Predict Hypermutated Cancers by Sequential Mutations

**Figure 5:**
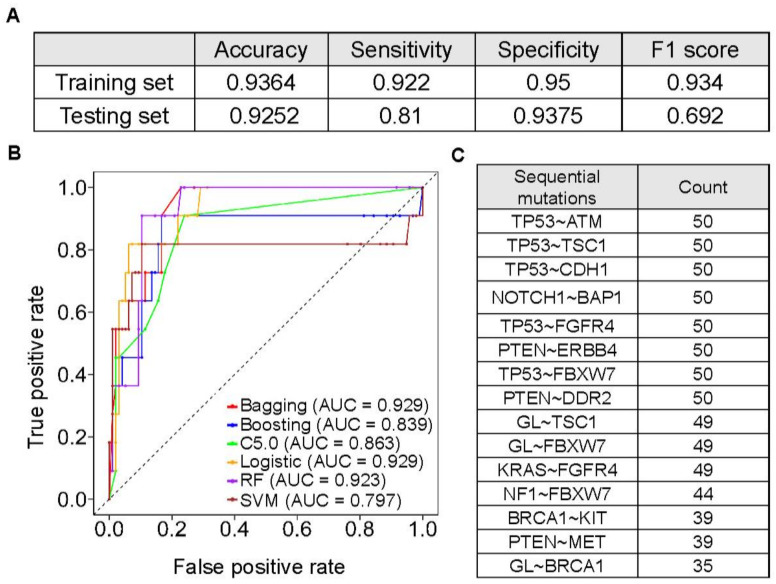
Hypermutated status prediction model. (**A**) A logistic regression model was used to predict hypermutations with the highest sensitivity. The table also shows the accuracy, specificity, and F1 scores for both the training (*n* = 755, 80%) and testing (*n* = 107, 20%) datasets. (**B**) Receiver operating characteristic (ROC) curves of six different classifiers and their area under the curve (AUC) values. Better sensitivity was achieved in the logistic regression model. RF, random forest; SVM, support vector machine. (**C**) Feature selection of hypermutation-associated sequential mutations. In total, fifteen sequential mutations were selected and are summarized in the table.

We next built a model to predict hypermutation status (Figure 5). We used the least absolute shrinkage and selection operator (LASSO) regression algorithm to select features of sequential mutations. To obtain more precise features, we implemented the algorithm 50 times and counted the frequency of the selected features. Only features that occurred more than 35 times were used for classification. The selected sequential mutations are listed in Figure 5C. To build a better classifier, we implemented six machine learning methods, including AdaBag (bagging), AdaBoost (boosting), C5.0, logistic regression (LR), random forest (RF), and support vector machine (SVM), for training (*n* = 755, 80%) and testing (*n* = 107, 20%). We used the oversampling technique in the training set. The area under the curve (AUC) was 0.93 in the bagging, RF, and LR models (Figure 5B and Figure S6). The logistic model had an accuracy of 0.93 and a sensitivity of 0.81 in the testing set (Figure 5A).

### 3.6. The Prevalence and Spectrum of Sequential Mutations

**Figure 6:**
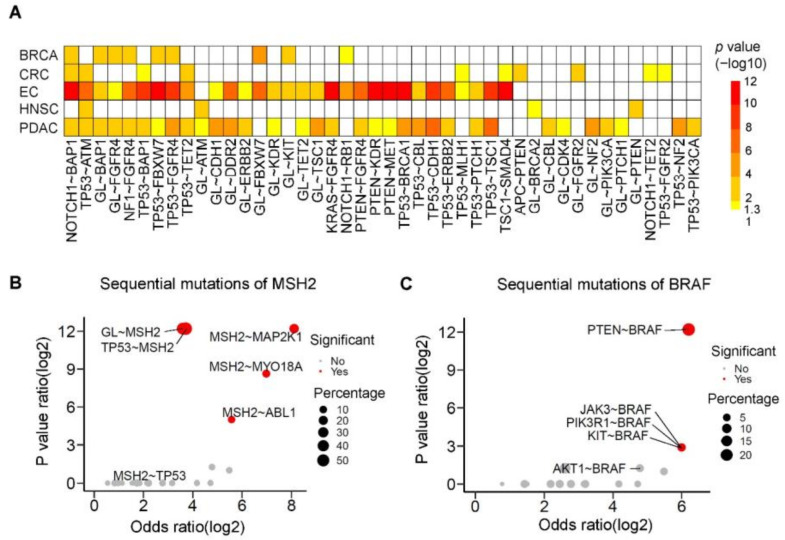
Hypermutation-associated sequential mutations. (**A**) Heatmap of hypermutation-associated sequential mutations in six cancer types. *p*-values were calculated by odds ratio with Bonferroni correction, and *p*-values > 0.05 are labeled in white. The most significant sequential mutations were found in endometrial and pancreatic cancer. For example, the *NOTCH1*~*BAP1* sequential mutation is observed in cancer patients. (**B**) Sequential mutations of *MSH2* by subgroup. The *TP53*~*MSH2* sequential mutation was associated with hypermutation, while the *MSH2*~*TP53* sequential mutation was not. *p*-values were calculated by odds ratio with Bonferroni correction, and *p*-values < 0.05 are labeled in red. The size of the dot represents the hypermutation percentage of the sequential mutation in all objects. (**C**) Sequential mutations of *BRAF* by subgroup. The *PTEN*~*BRAF*, *KIT*~*BRAF*, *PIK3R1*~*BRAF*, and *JAK3*~*BRAF* sequential mutations are associated with hypermutation, while the *AKT1*~*BRAF* sequential mutation is not.

Sequential cancer driver mutations could influence clinical presentation and response to therapy. We further investigated the prevalence and spectrum of sequential mutations in the six cancer types according to cancer evolution [21] (Table S9). The results are shown in Figure 6A and Figure S7. The observed sequential mutations differed by cancer type. EC and PDAC patients exhibited the greatest hypermutation-associated sequential mutations, whereas breast cancer, colorectal cancer, and ovarian cancer patients had fewer hypermutation-associated sequential mutations. For example, the most common hypermutation-associated sequential mutations were *NOTCH*~*BAP1* and *TP53*~*ATM*. CRC patients carried hypermutation-associated *TP53~MLH1* and *NOTCH1~TET2* sequential mutations. These findings may provide more genetic features for distinguishing hypermutation status or clinical outcome in hypermutated cancers.

The *MSH2* gene is a member of the DNA mismatch repair genes. *BRAF* gene mutation is correlated with *MLH1* gene methylation. Both are associated with hypermutation in cancer [33,34]. Data for *MSH2* and *BRAF* gene-associated sequential mutations are shown in Figure 6B,C. In our study, we found that sequential *MSH2* genetic mutations may affect hypermutation status. For example, the sequential genetic mutation of *TP53* followed by *MSH2* (*TP53*~*MSH2*) was observed in hypermutated cancers, but the mutation of *MSH2* followed by *TP53* (*MSH2*~*TP53*) was not (Figure 6B). Coexisting sequential mutations in *BRAF* also affected hypermutation status. *PETN*~*BRAF* sequential mutations were significantly more common than *AKT1*~*BRAF* sequential mutations in hypermutated cancers (Figure 6C). In addition to driver mutations, these results suggest that we need to consider sequential mutation order when analyzing hypermutation status.

### 3.7. Hypermutation-Associated Sequential Mutations and Clinical Outcome in Colorectal Cancer

**Figure 7:**
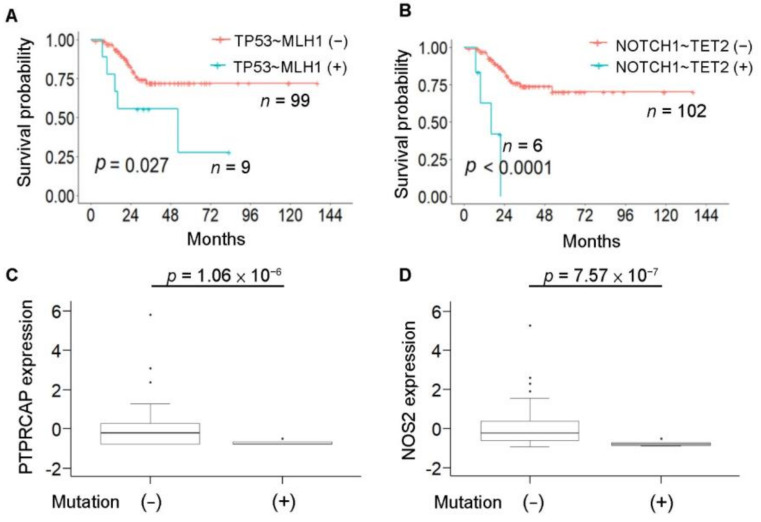
Hypermutation-associated sequential mutations impact survival. (**A**) Survival plot of *TP53~MLH1* hypermutation-associated sequential mutations in CRC patients. Patients without *TP53~MLH1* sequential mutations (*n* = 99) exhibited a better clinical outcome than patients with this set of mutations. *p*-value = 0.027 according to the log-rank test. (**B**) Survival plot of *NOTCH1*~*TET2* hypermutation-associated sequential mutations in CRC patients. Patients without *NOTCH1*~*TET2* sequential mutations (*n* = 102) exhibit a better clinical outcome than patients with this set of mutations. *p*-value < 0.05 by the log-rank test. (**C**) Boxplot of *NOTCH1*~*TET2* sequential mutations associated with immune gene expression with a *p*-value < 0.05. Patients with *NOTCH1*~*TET2* sequential mutations have reduced PTPRCAP expression compared to patients without this set of mutations. *p*-values were calculated by a *t*-test with Bonferroni correction. (**D**) Patients with *NOTCH1*~*TET2* sequential mutations exhibit reduced *NOS2* expression compared to patients without this set of mutations.

Here, we performed an analysis of the relationship among sequential mutations, immune gene expression, and clinical outcome in CRC. We found that hypermutation-associated *TP53*~*MLH1* and *NOTCH1*~*TET2* sequential mutations affected the survival of patients with colorectal cancer (*p*-values = 0.027 and 0.0001, respectively, Figure 7A,B). To study the relationship between sequential mutations and immune gene expression, tumor samples from 99 high-risk subjects were collected. After quality control, 78 subjects remained for further analysis. Patients with *NOTCH1*~*TET2* sequential mutations exhibited a lower expression of *PTPRCAP* (*p*-value = 1.06 × 10^−6^) and *NOS2* (*p*-value = 7.57 × 10^−7^), both of which are involved in the immune response, than patients with other sequential mutations (Figure 7C,D). Therefore, such sequential mutations may potentially represent hypermutation-associated prognostic biomarkers for stage III CRC patients.

## 4. Discussion

Although the FDA (US Food and Drug Administration) has approved immunotherapies for different hypermutated cancer types, there are still challenges related to drug resistance, therapeutic strategies, and biomarkers. Through comprehensive assessments of cancer gene panels, we investigated the heterogeneity of cancer driver mutations, mutational signatures, and evolution (intratumor) across different cancer types (intercancer) in hypermutated cancers. These advances have enhanced our ability to identify new immune targets and markers for the development of immunotherapy strategies. Here, we demonstrate the impact of cancer driver mutations on therapeutic strategies and clinical outcomes in hypermutated cancer patients. Our results highlight several important points. (i) There are heterogeneous driver mutations and mutational signatures in hypermutated cancers, implying that we should apply different therapeutic strategies for the different cancer types. (ii) *NF1* and *NOTCH1* neoantigens represent potential therapeutic targets for immunotherapy in hypermutated cancers. (iii) Sequential mutations, including different sequential orders and coexisting mutations, are good biomarkers for predicting hypermutated phenotypes and clinical outcomes. These findings suggest that the comprehensive analysis of mutational patterns in cancer, including driver mutations, mutational signatures, hypermutation-derived neoantigens, and sequential mutations, can have a significant impact on the care of hypermutated cancer patients.

We evaluated six specific cancer types. First, colorectal cancer and breast cancer are the most prevalent cancer types in the world. Next, according to official statistics, the incidence rate of gynecologic cancer, including endometrial and ovarian cancer, is increasing, and the diagnostic age in Taiwan has decreased in the past decade. The incidence rate of pancreatic cancer, which has the most unfavorable prognosis and survival rate, is also increasing in Taiwan. Finally, head and neck cancers have an incidence rate ten times greater in males than in females because of the habit of chewing betel nuts [35]. In a previous study, researchers demonstrated a significant correlation between TMB and the immune response across 27 solid tumor types [1]. Other studies also found that genetic mutations influenced the response to immunotherapy [36]. For example, *EGFR* and *STK11* mutations induce resistance to immunotherapy, while *MET* and *BRAF* genetic mutations induce sensitivity to immunotherapy [7,8].

Before immunotherapy, cancer should be checked for driver mutations and mutational signatures. For cancer driver mutations, most hypermutated PDAC patients carried *EGFR* mutations at a high frequency, which explains their poorer clinical response to immunotherapy [1]. Most hypermutated HNSC patients carrying *ATM* mutations may have a better clinical response to immunotherapy than those without *ATM* mutations [37]. Most hypermutated EC patients carrying *MSH2* gene mutations may have a better clinical response than those without *MSH2* mutations [38]. Defective DNA replication repairs resulting in germline mutations are associated with hypermutations in CRC or OC [8]. Testing for cancer genomic profiling is associated with the identification of germline pathogenic variants. For OC patients with BRCA1/2 cancer driver mutations, PARP inhibitors are the standard of care rather than immunotherapy. Genetic counseling of the relatives of cancer patients is essential [39]. In addition, we identified *RET*, *CBL*, and *DDR2* driver mutations as potential response markers in hypermutated cancers. In our study, we provide additional, comprehensive driver genetic mutations and response markers in immunotherapy. However, further studies are needed to obtain related biological information.

Mutational signatures can reflect the presence or absence of cellular processes in cancer cells. In addition to environmental factors (such as UV light, tobacco smoking, and alkylating agents), intrinsic sources, such as *APOBEC* and DNA repair genes, have been described as the major cause of hypermutation in cancer. In this study, the relationship between signatures 2/11 and 30 was extremely positively correlated with hypermutation status. Age, alkylating agent exposure, DNA repair defects, and *APOBEC* mutations are drivers of mutational signatures in hypermutated cancers. Beyond the cancer driver mutations, DNA repair defects and *APOBEC* mutational signatures are good makers for immunotherapy responses.

Tumor neoantigens are essential for personalized immunotherapy. We showed a relationship between TMB and neoantigen numbers. For neoantigen prediction, we used the Taiwan population database to simulate and calculate HLA-A, HLA-B, and HLA-C binding affinity and found two potential neoantigens, *NF1* p.T700I and *NOTCH1* p.V2153M. These neoantigens can contribute to the development of vaccines and cell-based therapeutic strategies, especially in Asian populations.

In this study, we developed a new concept for hypermutated cancers based on sequential mutations. Aside from cancer driver mutations, sequential mutations are very important for hypermutated cancers. Identifying cancer types based on cancer driver mutation data is still a challenge. To address this, we built a tumor evolution model to distinguish hypermutated cancers from nonhypermutated cancers. We showed that sequential mutations could distinguish hypermutated cancer patients, whereby tumor evolution is analyzed via hierarchical clustering of genetic driver mutations and clonality. Sequential mutations also accurately predicted hypermutation status. Beyond the driver mutations, the sequential mutation order of *MSH2* and coexisting *BRAF* genetic mutations influence patients with a hypermutated cancer phenotype. For the clinical impact of sequential mutations, we found that the *NOTCH1*~*TET2* sequential mutations correlated with reduced *PTPRCAP* expression and that *NOS2* expression was associated with a worse outcome than a lack of *NOS2* expression [40]. In a previous study, mice with both *NOTCH1* and *TET2* gene inactivation also exhibited lower survival [41]. These results imply that sequential mutations are potential predictive markers for the clinical impact of hypermutated cancers.

Our study has some limitations. False-negative and false-positive mutations due to NGS errors contribute to inconsistencies in bioinformatic analyses. Many studies have demonstrated filtering common germline polymorphisms from tumor-only NGS data by population frequency [12]. However, true somatic mutations should be identified by tumor and blood samples. One disadvantage of FFPE is the use of formalin, which may cause sequence artifacts. In a previous study, 63–70% of the variants in the fresh-frozen (FF) exome sequencing datasets were also found in the FFPE exome sequencing datasets. The FFPE and FF samples have similar spectra of mutations based on the trinucleotide content compared with 30 signatures from the COSMIC database by hierarchical clustering [42]. Deamination effects do not lead to false-positive mutations in clinical practice [43]. In addition, since low-coverage samples have higher rates of deamination effects (C > T/G > A sequencing artifacts) in formalin fixation [44], we used deep targeted sequencing (average coverage > 1000×) technology to process our samples.

## 5. Conclusions

We conducted a comprehensive analysis of mutational patterns, including sequential mutations, and identified useful markers and therapeutic targets for application in the therapy of hypermutated cancer patients.

## Data Availability

The datasets used and analyzed during the current study are available from the corresponding author on reasonable request, and supplementary information files are available for this manuscript.

## Supplementary Materials

The following are available online at https://www.mdpi.com/article/10.3390/cancers13174317/s1, Figure S1: Genetic variants of breast (BRCA), colorectal (CRC), endometrial (EC), oral (HNSC), ovarian (OV), and pancreatic (PDAC) cancer., Figure S2: The correlation of tumor mutational burden with mutated genes identified by whole-exome sequencing (WES) and the Oncomine cancer panel., Figure S3: COSMIC mutation signatures in cancer patients., Figure S4: Analysis of neoantigen binding to HLA alleles., Figure S5: Repeated evolutionary trajectories of sequential mutations in our cohort., Figure S6: Table showing the performance of six different classifiers in the training and testing sets for hypermutation prediction., Figure S7: Sequential mutations by group. Hypermutation-associated sequential mutations of breast cancer (BRCA), pancreatic cancer (PDAC), oral cancer (HNSC), colorectal cancer (CRC), endometrial cancer (EC), and all 6 cancer types., Table S1: The spectra and frequencies of driver mutations in different cancers., Table S2: Driver mutations in hypermutated cancer., Table S3: The frequency and similarity of gene mutations in TCGA samples., Table S4: Driver mutations based on specific histology types in hypermutated endometrial and ovarian cancer., Table S5: Driver mutations in different hypermutated cancers., Table S6: The correlation of exonic variants and neoantigen number., Table S7: Hypermutation-associated neoantigens., Table S8: Hypermutation-associated sequential mutations., Table S9: Hypermutation-associated sequential mutations in different cancer types.

## Abbreviations

AUC	area under the curve
APOBEC	apolipoprotein B mRNA editing enzyme, catalytic polypeptide-like
BRCA	breast cancer
BF	Bonferroni
CRC	colorectal cancer
COSMIC	Catalog of Somatic Mutations in Cancer
EC	endometrial cancer
ECOG PS	Eastern Cooperative Oncology Group performance status
ExAC-EAS	Exome Aggregation Consortium-East Asian
FDA	U.S. Food and Drug Administration
FDR	false discovery rate
FF	fresh-frozen
FFPE	formalin-fixed paraffin-embedded
HNSC	head and neck cancer
ICI	immune checkpoint inhibitor
LASSO	least absolute shrinkage and selection operator
LR	logistic regression
MSI-H	microsatellite instability-high; mut/Mb: mutations per megabase
NCKUH	National Cheng Kung University Hospital
NGS	next generation sequencing
OV	ovarian cancer
PCA	principal component analysis
RFS	recurrence-free survival
PDL-1	programmed death-ligand 1
PDAC	pancreatic cancer
RF	random forest
SNVs	single-nucleotide variants
SVM	support vector machine
TAF	Taiwan Accreditation Foundation
TCGA	The Cancer Genome Atlas
TFDA-LDTs	Taiwan Food and Drug Administration
TMB	tumor mutational burden
WES	whole-exome sequencing
