# Supplementary material for: Comprehensively Exploring the Mutational Landscape and Patterns of Genomic Evolution in Hypermutated Cancers
Source: Cancers (Basel). 2021 Aug 26;13(17):4317. doi: 10.3390/cancers13174317 (PMC8431047; doi:10.3390/cancers13174317)
Supplement: Supplementary file 1 [file cancers-13-04317-s001.zip › cancers-1349781 supplementary figure.pdf]

cancers

Supplementary Material: Comprehensively exploring the mutational landscape and patterns of genomic evolution in hypermutated cancers

Peng-Chan Lin, Yu-Min Yeh, Hui-Ping Hsu, Ren-Hao Chan, Bo-Wen Lin, Po-Chuan Chen, Chien-Chang Pan, Keng-Fu Hsu, Jenn-Ren Hsiao, Yan-Shen Shan and Meng-Ru Shen

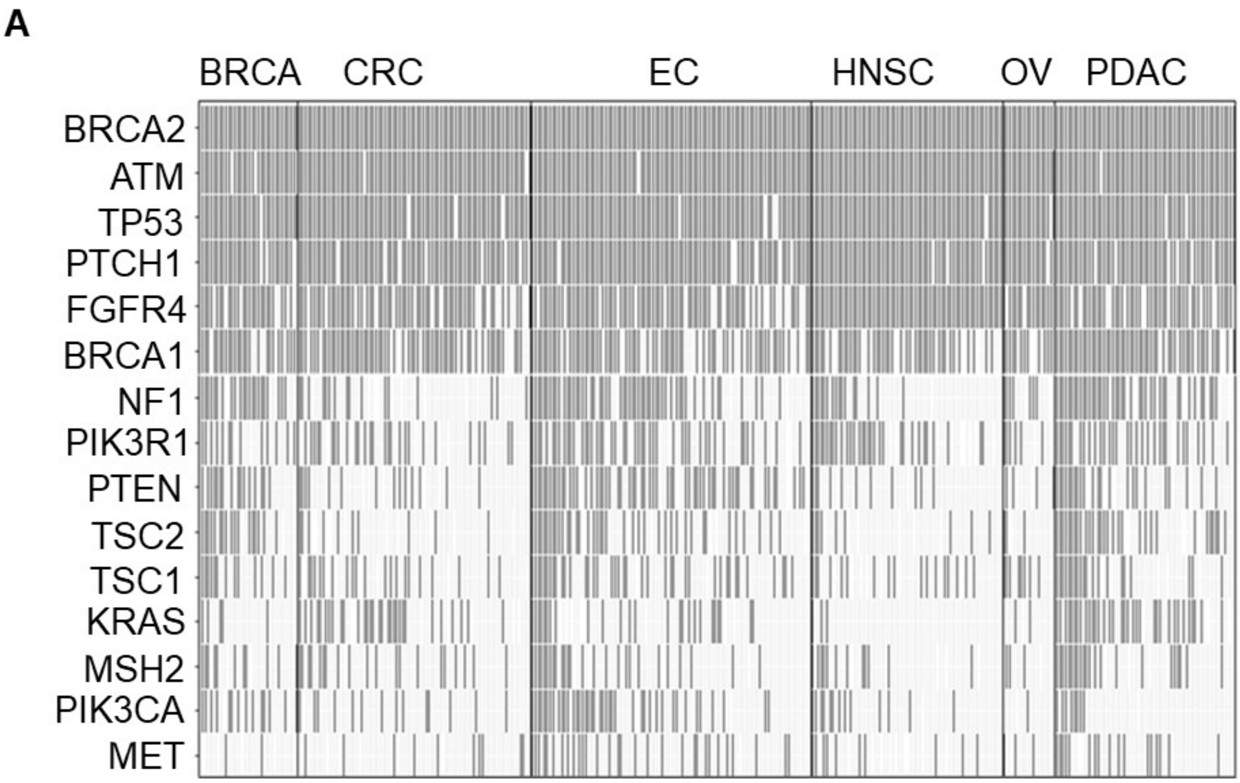

Figure S1. Genetic variants of breast (BRCA), colorectal (CRC), endometrial (EC), oral (HNSC), ovarian (OV) and pancreatic (PDAC) cancer. a. Heatmap of the variants in the 6 cancer types. The top 15 genetic variants are shown in the heatmap, and BRCA2 was the most frequently altered gene.

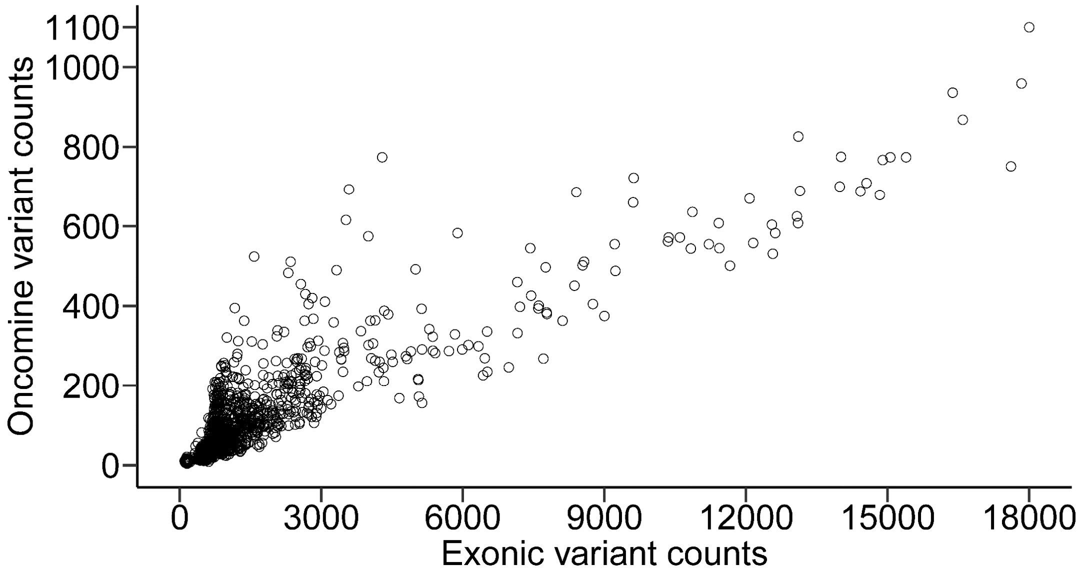

Figure S2. The correlation of tumor mutational burden with mutated genes identified by whole-exome sequencing (WES) and the Oncomine cancer panel. A high correlation was noted (*R*^2=0.82) (TCGA colorectal cancer dataset).

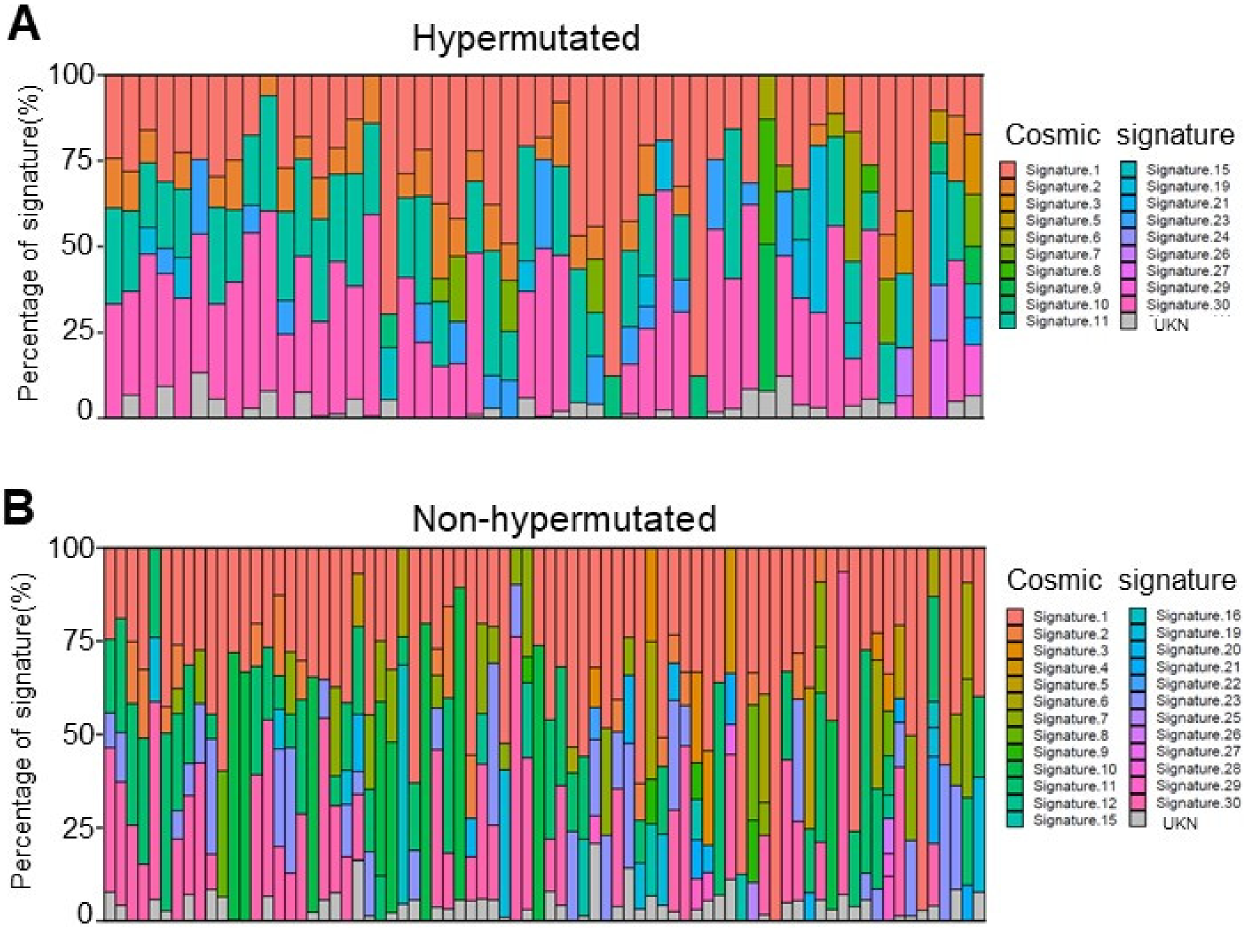

Figure S3. COSMIC mutation signatures in cancer patients. (**A**) Bar plot of patients with hypermutated cancer and their percentages of different mutation signatures. Signature NA indicates an unknown component. (**B**) Bar plot of patients with nonhypermutated cancer and their percentages of different mutation signatures. Signature NA indicates an unknown component.

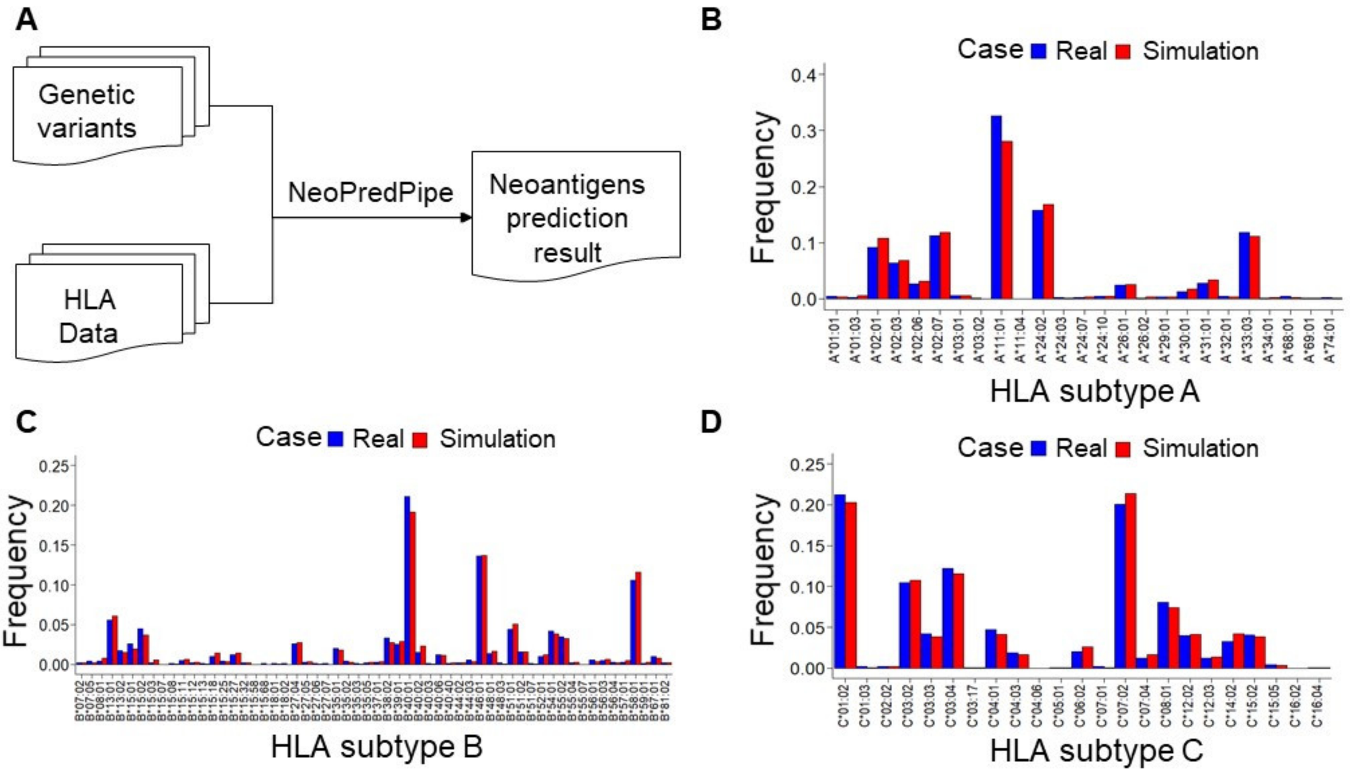

Figure S4. Analysis of neoantigens binding to HLA alleles. (**A**) Pipeline of neoantigen prediction. We analyzed the genetic variants and HLA data to predict possible neoantigens and their binding affinities with HLA-A, HLA-B and HLA-C molecules. (**B**) Simulation results for HLA-A. Blue and red bars indicate distribution of the Chinese population and simulation frequency, respectively. (**C**) Simulation results for HLA-B. (**D**) Simulation results for HLA-C.

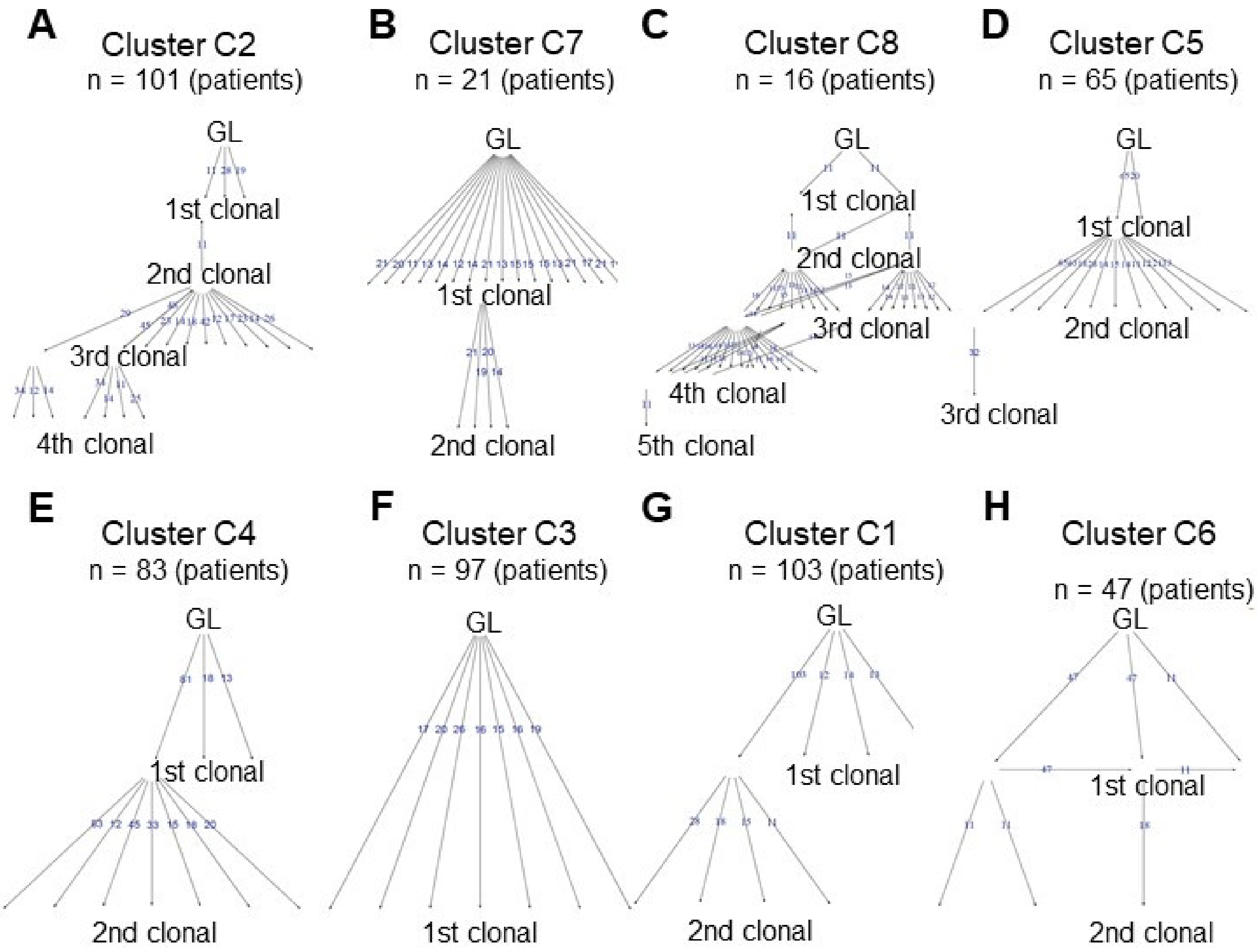

Figure S5. Repeated evolutionary trajectories of sequential mutations in our cohort. The 8 clusters were detected by REVOLVER. A-H: Graph showing annotation of group size (n) and trajectory number of times on the edge (>10 times). A-C: Hypermutated pattern. D-H: Nonhypermutated pattern. (**A**) Cluster C2 first clone drivers: FBXW7, KRAS and PIK3CA; second clone drivers: TP53, APC, ATM, BAP1, BRCA1, NF1, PIK3R1, RB1, TET2, TSC1 and TSC2; and third clone drivers: BRCA2, CDH1, VHL, PTEH, MSH2, NF2 and PTCH1. (**B**) Cluster C7 with first clone drivers: NF1, ATM, BAP1, BRCA1, FBWX7, KDR, KRAS, TP53, PIK3CA, PTCH1, PTEN, RB1, STK11, TET2, TSC1, TSC2 and VHL; and second clone drivers: APC, BRCA2, CDH1 and NOTCH1. (**C**) Cluster C8 with first clone drivers: CDKN2A and PIK3R1; second clone drivers: NOTCH1 and PTEN; third clone drivers: TP53, KRAS, NF1, BAP1, MAP2K1, PDGFRA, PRAG, RB1, RET, SMAD4, APC, CTNNB1, EGFR, ERBB4, KDR, MET and WT1; fourth clone drivers: FBXW7, BRCA2, BRCA1, MSH2, TSC1, ATM, CDH1, FGFR4, FLT3, NF2, PIK3CA, PTCH1, TET2 and TSC2; and fifth clone driver: GATA3. (**D**) Cluster C5 with first clone drivers: TP53 and KRAS; second clone drivers: NOTCH1, APC, ATM, BRCA2, MSH2, PIK3CA, PTCH1, PTEN, RET, TSC2 and VHL; and third clone driver: NF1. (**E)** Cluster C4 with first clone drivers: TP53, KRAS and PIK3CA; and second clone drivers: APC, ATM, BRCA2, NF1, PTCH1, PTEN and TSC2. (**F**) Cluster C3 with first clone drivers: KRAS, NF1, NOTCH1, PIK3CA, PTEN, STK11 and VHL. (**G**) Cluster C1 first clone drivers: TP53, KRAS, PIK3CA and TSC2; and second clone drivers: BRCA2, NF1, PTEN and TSC1. (**H**) Cluster C6 first clone drivers: TP53, NOTCH1 and KRAS; and second clone drivers: BRCA2, TSC2 and NF1.

Training n=755

Model	Accuracy	Sensitivity	Specificity	F1 score
SVM	0.947	0.903	0.987	0.944
C5.0 tree	0.9497	0.922	0.976	0.947
Random Forest	0.9457	0.903	0.987	0.943
Bagging	0.9139	0.89	0.937	0.91
Boosting	0.955	0.922	0.987	0.952
LR	0.9364	0.922	0.95	0.934

Testing n=107

Model	Accuracy	Sensitivity	Specificity	F1 score
SVM	0.9346	0.54	0.979	0.631
C5.0 tree	0.9159	0.45	0.9687	0.526
Random Forest	0.8972	0.36	0.9583	0.421
Bagging	0.8598	0.63	0.8854	0.56
Boosting	0.9065	0.45	0.9583	0.5
LR	0.9252	0.81	0.9375	0.692

Figure S6. Table showing the performance of 6 different classifiers in the training and testing sets for hypermutation prediction. The table shows the accuracy, sensitivity, specificity and F1 score of the SVM, C5.0, random forest, bagging, boosting and logistic regression models. Logistic regression (LR) had the best sensitivity and F1 score in the testing set. We built our machine learning model using LR.

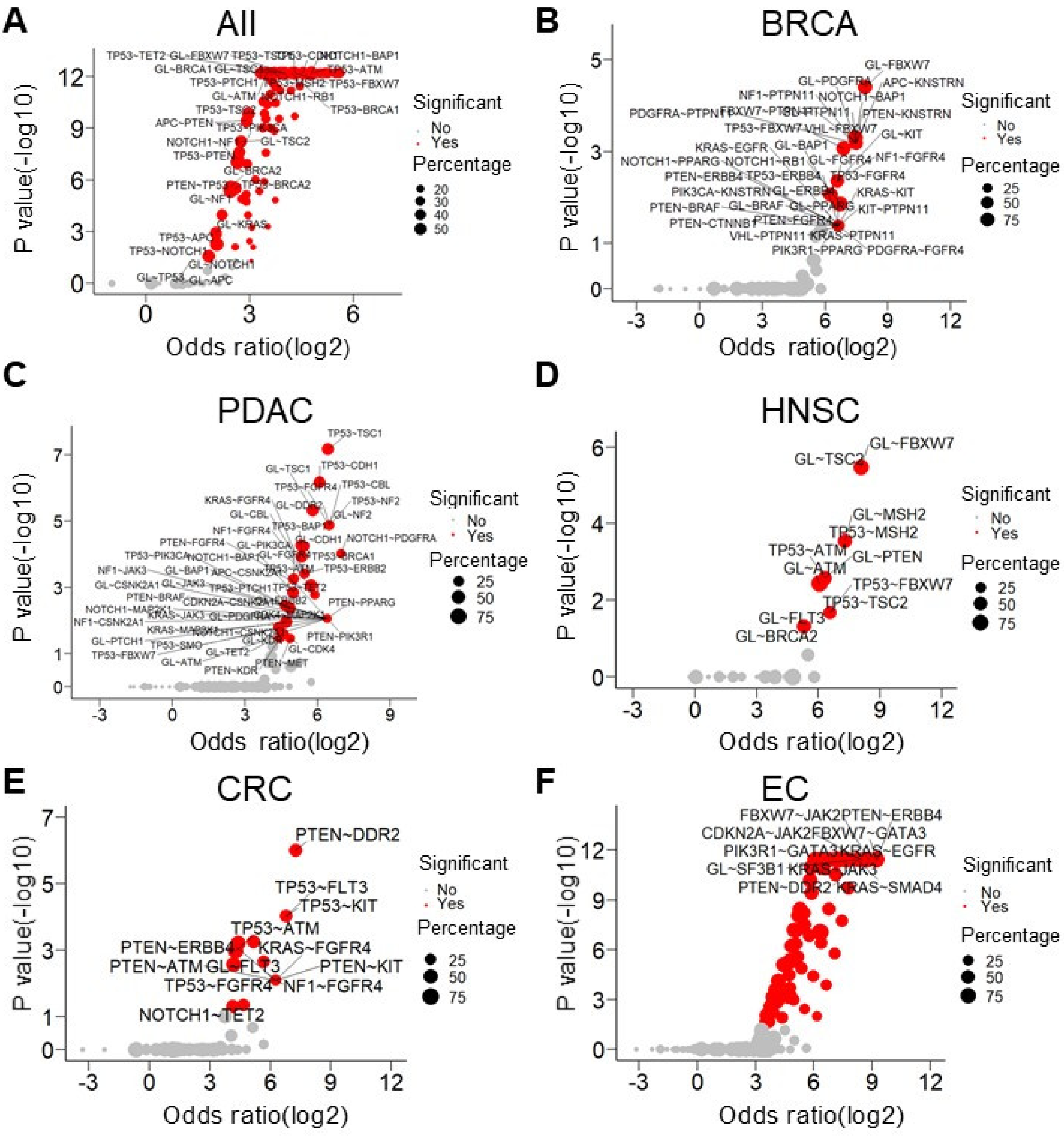

Figure S7. Sequential mutations by group. Hypermutation-associated sequential mutations of breast cancer (BRCA), pancreatic cancer (PDAC), oral cancer (HNSC), colorectal cancer (CRC), endometrial cancer (EC) and all 6 cancer types. (**A**) Top 30 hypermutation-associated sequential mutations in all cancer types. The P-value was calculated by an odds ratio with a Bonferroni correction, and a p-value less than 0.05 is labeled with a red color. The size of the dot shows the percentage of hypermutated objects with phylogenetic mutations. (**B**) There were 34 hypermutation-associated sequential mutations in BRCA. (**C**) There were 50 hypermutation-associated sequential mutations in PDAC. (**D**) There were 11 hypermutation-associated sequential mutations in HNSC. (**E**) Top 10 hypermutation-associated sequential mutations in CRC. (**F**) Top 10 hypermutation-associated sequential mutations in EC.
